# Creatine, guanidinoacetate and homoarginine in statin-induced myopathy

**DOI:** 10.1007/s00726-020-02865-w

**Published:** 2020-06-27

**Authors:** Axel Neu, Sönke Hornig, Ali Sasani, Dirk Isbrandt, Christian Gerloff, Dimitris Tsikas, Edzard Schwedhelm, Chi-un Choe

**Affiliations:** 1 Experimental Neuropediatrics, Department of Pediatrics, University Medical Center Hamburg-Eppendorf, Hamburg, Germany; 2 Department of Neurology, University Medical Center Hamburg-Eppendorf, Martinistraße 52, 20246 Hamburg, Germany; 3 German Center for Neurodegenerative Diseases (DZNE), Bonn, Germany; 4 University of Cologne, Cologne, Germany; 5 Core Unit Proteomics, Hannover Medical School, Institute of Toxicology, Hannover, Germany; 6 Institute of Clinical Pharmacology and Toxicology, University Medical Center Hamburg-Eppendorf, Hamburg, Germany; 7 German Center for Cardiovascular Research (DZHK), Partner Site Hamburg/Kiel/Lübeck, Hamburg, Germany

## Abstract

Our study evaluated the effect of creatine and homoarginine in AGAT- and GAMT-deficient mice after simvastatin exposure. Balestrino and Adriano suggest that guanidinoacetate might explain the difference between AGAT- and GAMT-deficient mice in simvastatin-induced myopathy. We agree with Balestrino and Adriano that our data shows that (1) creatine possesses a protective potential to ameliorate statin-induced myopathy in humans and mice and (2) homoarginine did not reveal a beneficial effect in statin-induced myopathy. Third, we agree that guanidinoacetate can be phosphorylated and partially compensate for phosphocreatine. In our study, simvastatin-induced damage showed a trend to be less pronounced in GAMT-deficient mice compared with wildtype mice. Therefore, (phospo) guanidinoacetate cannot completely explain the milder phenotype of GAMT-deficient mice, but we agree that it might contribute to ameliorate statin-induced myopathy in GAMT-deficient mice compared with AGAT-deficient mice. Finally, we agree with Balestino and Adriano that AGAT metabolites should further be evaluated as potential treatments in statin-induced myopathy.

Dear editor,

We thank Drs. Balestrino and Adriano for their insightful comments on our publication about the effects of AGAT- and GAMT-deficiency in simvastatin-induced myopathy (Balestrino and Adriano 2020). Among all our findings, Balestrino and Adriano point out the increased vulnerability of simvastatin induced myopathy in AGAT-deficient (AGAT^−/−^) mice compared with wildtype and GAMT-deficient (GAMT^−/−^) mice (Sasani et al. 2020). AGAT^−/−^ mice are devoid of creatine, homoarginine and guanidinoacetate—the only known products of the AGAT and GAMT pathway.

First, we have shown that creatine reduces simvastatin-induced muscle damage in creatine-deficient AGAT^−/−^ mice. This finding in mice is in line with their and other previous work in patients (Balestrino and Adriano 2018; Shewmon and Craig 2010). In mice, we have shown that creatine-deficient AGAT^−/−^ and GAMT^−/−^ mice reveal a severe myopathy and reduced muscle strength (Nabuurs et al. 2013; Schmidt et al. 2004). Therefore, creatine possesses a protective potential to ameliorate statin-induced myopathy in humans and mice.

Second, homoarginine was studied in statin-induced myopathy, given that we were the first to show that AGAT is mandatory for homoarginine synthesis in mice and humans (Choe et al. 2013). However, our current experiments revealed that homoarginine supplementation in homoarginine-deficient AGAT^−/−^ mice did not affect simvastatin-induced myopathy, which is in contrast to homoarginine’s role in cerebrovascular and cardiac function (Choe et al. 2013; Faller et al. 2018). In mouse models of ischemic stroke and heart failure, homoarginine—but not creatine—was able to improve cerebrovascular damage and normalize cardiac dysfunction in AGAT-deficient mice. We agree with Balestrino and Adriano that although we hypothesized a protective effect of homoarginine in statin-induced myopathy, we did not find any.

The third product of AGAT activity is guanidinoacetate. GAMT-deficient mice are devoid of creatine—as AGAT-deficient mice, but have increased AGAT expression and, therefore, higher guanidinoacetate levels. Therefore, Balestrino and Adriano hypothesized that the milder phenotype of GAMT- versus AGAT-deficient mice might rather be explained by elevated guanidinoacetate levels and not AGAT expression itself. Early studies with GAMT-deficient mice and patients revealed that guanidinoacetate can also be phosphorylated in the absence of creatine to partially substitute for phosphocreatine (Kan et al. 2004; Schulze et al. 1997). However, enzyme kinetics and recovery rates after depletion of phospho-guanidinoacetate are considerably slower compared to phosphocreatine. Phospho-guanidinoacetate is not able to fully compensate for the lack of phosphocreatine, because GAMT-deficient mice reveal a reduced muscle strength and histological signs of myopathy (Schmidt et al. 2004). Moreover, in addition to AGAT- and GAMT-deficient mice, we also used wild-type controls in our experiments with normal creatine, normal homoarginine and normal guanidinoacetate levels (Sasani et al. 2020). Interestingly, statin-induced muscle damage and dysfunction showed a trend to be less pronounced in GAMT-deficient mice compared with wild-type mice. Although phospho-guanidinoacetate cannot completely explain the milder phenotype of GAMT-deficient mice, (phosho) guanidinoacetate might contribute to ameliorate statin-induced myopathy in GAMT-deficient mice compared with AGAT-deficient mice as suggested by Balestrino and Adriano (Balestrino and Adriano 2020). In addition to their protective roles as energy buffers, (phospho) guanidinoacetate might have similar pleitropic effects as (phospho) creatine (Wallimann et al. 2011), such as being an osmolyte that protects muscle against exercise-induced hypertonic stress (Alfieri et al. 2006). Finally, we agree with Balestino and Adriano that AGAT metabolites should further be evaluated as potential treatments in statin-induced myopathy.
